# Improvement of cerebellar ataxic gait by injecting Cbln1 into the cerebellum of *cbln1*-null mice

**DOI:** 10.1038/s41598-018-24490-0

**Published:** 2018-04-18

**Authors:** Eri Takeuchi, Aya Ito-Ishida, Michisuke Yuzaki, Dai Yanagihara

**Affiliations:** 1 Department of Life Sciences, Graduate School of Arts and Sciences, The University of Tokyo, 3-8-1 Komaba, Meguro, Tokyo, 153-8902 Japan; 2 Department of Physiology, School of Medicine, Keio University, 35 Shinanomachi, Shinjuku, Tokyo, 160-8582 Japan

## Abstract

Patients and rodents with cerebellar damage display ataxic gaits characterized by impaired coordination of limb movements. Here, gait ataxia in mice with a null mutation of the gene for the cerebellin 1 precursor protein (*cbln1*-null mice) was investigated by kinematic analysis of hindlimb movements during locomotion. The Cbln1 protein is predominately produced and secreted from cerebellar granule cells. The cerebellum of *cbln1*-null mice is characterized by an 80% reduction in the number of parallel fiber-Purkinje cell synapses compared with wild-type mice. Our analyses identified prominent differences in the temporal parameters of locomotion between *cbln1*-null and wild-type mice. The *cbln1*-null mice displayed abnormal hindlimb movements that were characterized by excessive toe elevation during the swing phase, and by severe hyperflexion of the ankles and knees. When recombinant Cbln1 protein was injected into the cerebellum of *cbln1*-null mice, the step cycle and stance phase durations increased toward those of wild-type mice, and the angular excursions of the knee during a cycle period showed a much closer agreement with those of wild-type mice. These findings suggest that dysfunction of the parallel fiber-Purkinje cell synapses might underlie the impairment of hindlimb movements during locomotion in *cbln1*-null mice.

## Introduction

The cerebellum has a crucial role in the control of posture and locomotion^1^. The importance of the cerebellum is illustrated by the impaired posture and locomotion shown by cerebellar patients^1,2^ who often exhibit a lack of coordination of limb movements^3–5^. In a previous study, we showed that mice with a mutation of the *hotfoot* gene (*ho15J*) had cerebellar defects and an ataxic gait^6^. During treadmill locomotion, the *ho15J* mice had an abnormally flexed joint angle in their hindlimbs. They also showed incoordinate limb joint movements^6^. The *ho15J* mutation is the result of an intragenic deletion in a gene encoding the δ2 glutamate receptor (GluD2)^7^. GluD2 mutant mice (including GluD2-null and *hotfoot*) have a decreased number of parallel fiber (PF)-Purkinje cell (PC) synapses, multiple climbing fiber (CF) innervation, and impaired long-term depression^8–10^. The GluD2 protein was recently identified as a postsynaptic receptor of Cbln1^11^, while neurexin (Nrx) has been identified as a presynaptic receptor of Cbln1^12^. Cbln1 is a glycoprotein that belongs to the C1q family. It is predominantly secreted from synaptic ends of PFs in the cerebellum^13^ and is essential for the establishment or maintenance of PF-PC synapses^14,15^. Cbln1 induces dynamic structural changes in PFs through Nrx-Cbln1-GluD2 signaling^16^. The role of the protein has been demonstrated in *cbln1*-null mice, which show gait ataxia, a severely reduced number of PF-PC synapses, an impaired performance on an accelerating rotarod, and an irregular gait pattern^14^. A previous study reported that application of a recombinant Cbln1 protein to the adult cerebellum restored synapse formation between PFs and PCs in the adult cerebellum, and improved locomotive performance so that gait pattern and behavior on an accelerating rotarod apparently returned to normal^15^. At present, it is unclear how PF-PC synapses act in locomotion. To investigate the function of PF-PC synapses, we compared the hindlimb movements of adult *cbln1*-null mice before and after injection of recombinant Cbln1.

## Results

Wild-type mice were able to walk on the treadmill at all tested speeds, but *cbln1*-null mice were unable to walk stably and continuously when the speed was set to 16 or 24 m/min. As a result, *cbln1*-null mice frequently fell off the treadmill. Furthemore, we observed that the *cbln1*-null mice more frequently lacked a bisupport phase when compared to wild-type mice. Therefore, we restricted the following analyses to a treadmill speed of 8 m/min. We used averages of pooled data for wild-type and *cbln1*-null mice for the comparisons, unless otherwise stated. We pooled both male and female mice and we observed no significant difference between males and females.

### Characteristics of ataxic gait in the *cbln1*-null mice

**Figure 1:**
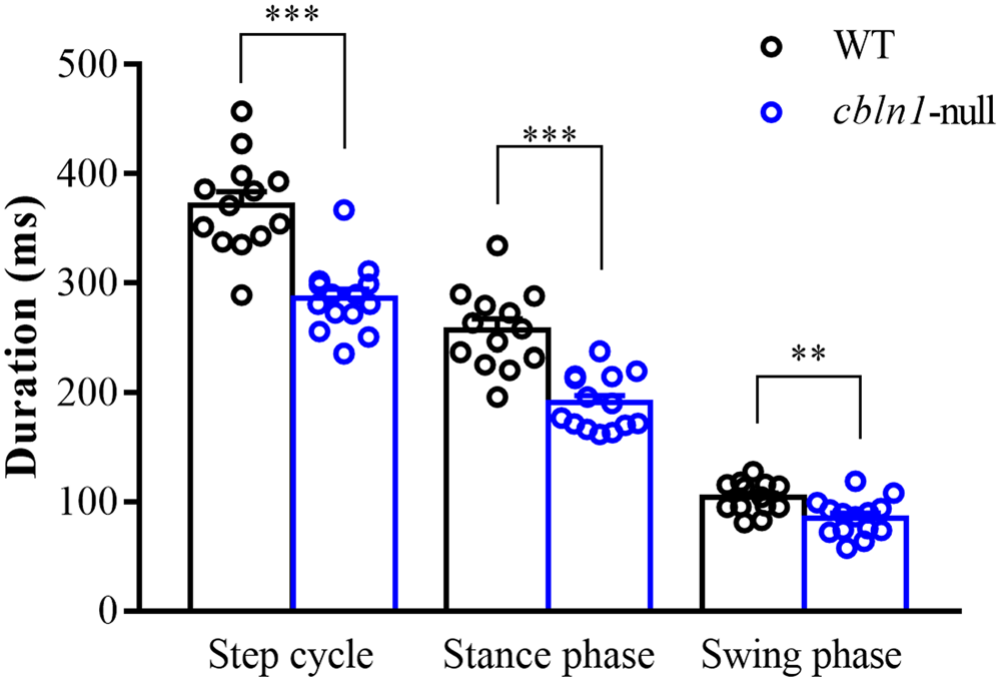
Average step cycle duration. The histograms display average durations of overall step cycle, stance, and swing phases in wild-type (WT) and *cbln1*-null mice. **p < 0.01; ***p < 0.001.

The average durations of the step cycle, stance phase, and swing phase are shown in Fig. 1. The duration of each phase of the step cycle was significantly shorter in *cbln1*-null mice than in wild-type mice (step: *cbln1*-null (n = 14) = 286.0 ± 8.4 ms, wild-type (n = 13) = 371.5 ± 12.1 ms, t (25) = 5.873, *p* < 0.001; stance: *cbln1*-null = 190.6 ± 6.7 ms, wild-type = 257.2 ± 10.1 ms, t (25) = 5.550, *p* < 0.001; swing: *cbln1*-null = 85.4 ± 4.5 ms, wild-type = 104.3 ± 4.0 ms, t (25) = 3.107, *p* < 0.01). There was a positive correlation between duration and body weight when all individuals were compared together, but there was no correlation when each genotype was compared separately (Supplementary Fig. 1).

**Figure 2:**
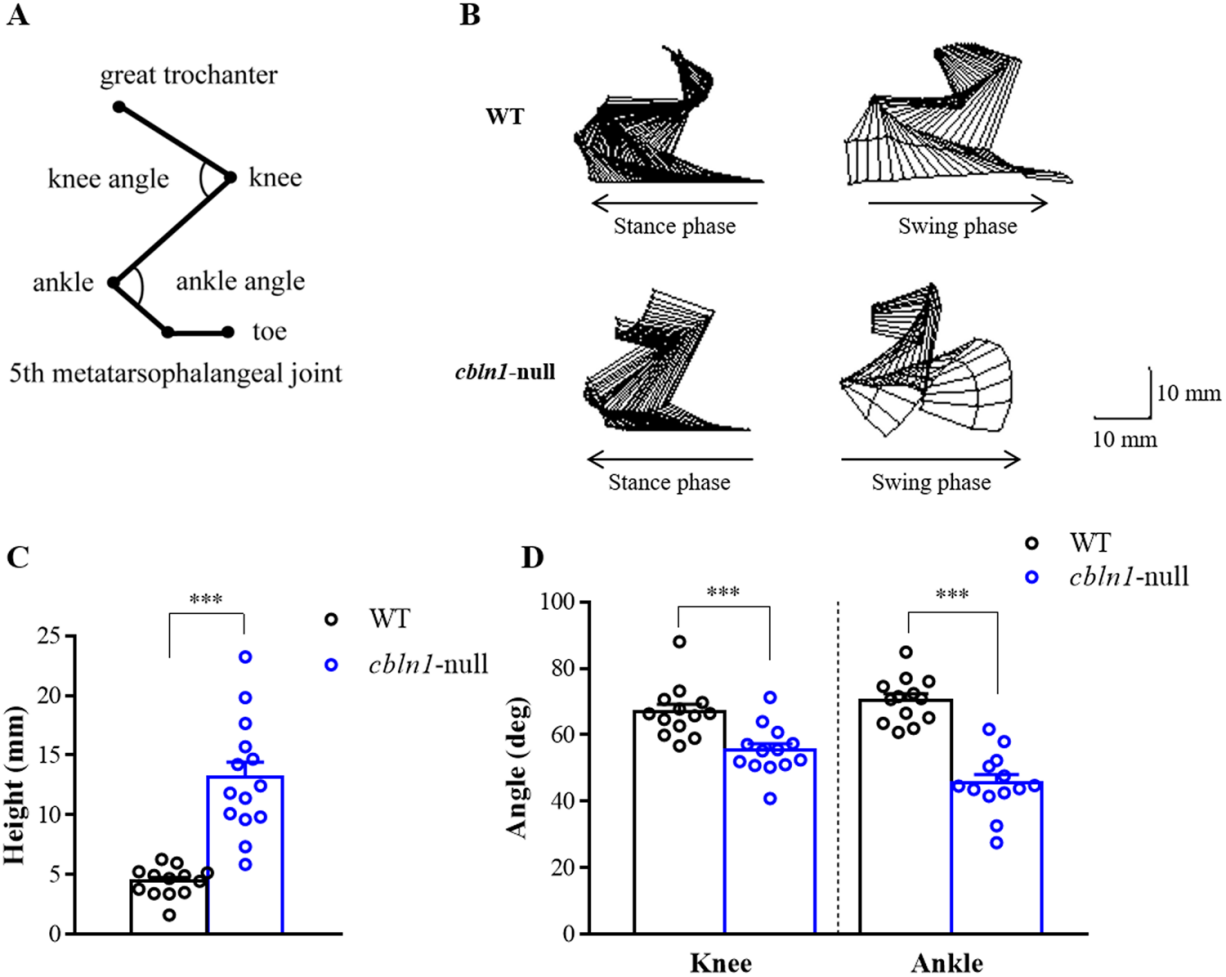
Kinematics of maximal toe height during the step cycle. (**A**) Positions of the reflective markers. Reflective markers were placed on the following anatomical landmarks: great trochanter, knee, ankle, 5th metatarsophalangeal (MTP) joint, and a toe. (**B**) Typical examples of stick figure representations of stance and swing phases for a wild-type (WT) and a *cbln1*-null mouse. Scale bar indicates 10 mm. (**C**) Maximal toe heights in wild-type and *cbln1*-null mice. (**D**) Joint angles at maximal toe heights. ***p < 0.001.

Stick figure representations of the hindlimbs during treadmill locomotion are presented in Fig. 2B; for the right hindlimb, stick figures for the one step cycle show the stance and swing phases separately. The hindlimbs of *cbln1*-null mice had different trajectories to those of wild-type mice. In particular, the *cbln1*-null mice showed excessive toe elevation during the swing phase. We measured the maximal toe height of *cbln1*-null mice (Fig. 2C) and found that during the swing phase, it was significantly higher than in wild-type mice (*cbln1*-null = 13.1 ± 1.3 mm, wild-type = 4.4 ± 0.3 mm, t (14.86) = 6.534, *p* < 0.001). Knee and ankle angles in *cbln1*-null mice were significantly lower than in wild-type mice (knee: *cbln1*-null = 55.3 ± 2.1 deg, wild-type = 67.0 ± 2.2 deg, t (24) = 3.906, *p* < 0.001; ankle: *cbln1*-null = 45.5 ± 2.5 deg, wild-type = 70.4 ± 1.9 deg, t (24) = 7.851, *p* < 0.001, Fig. 2D). These results indicated that *cbln1*-null mice showed excessive toe elevation due to hyperflexion of knees and ankles.

**Figure 3:**
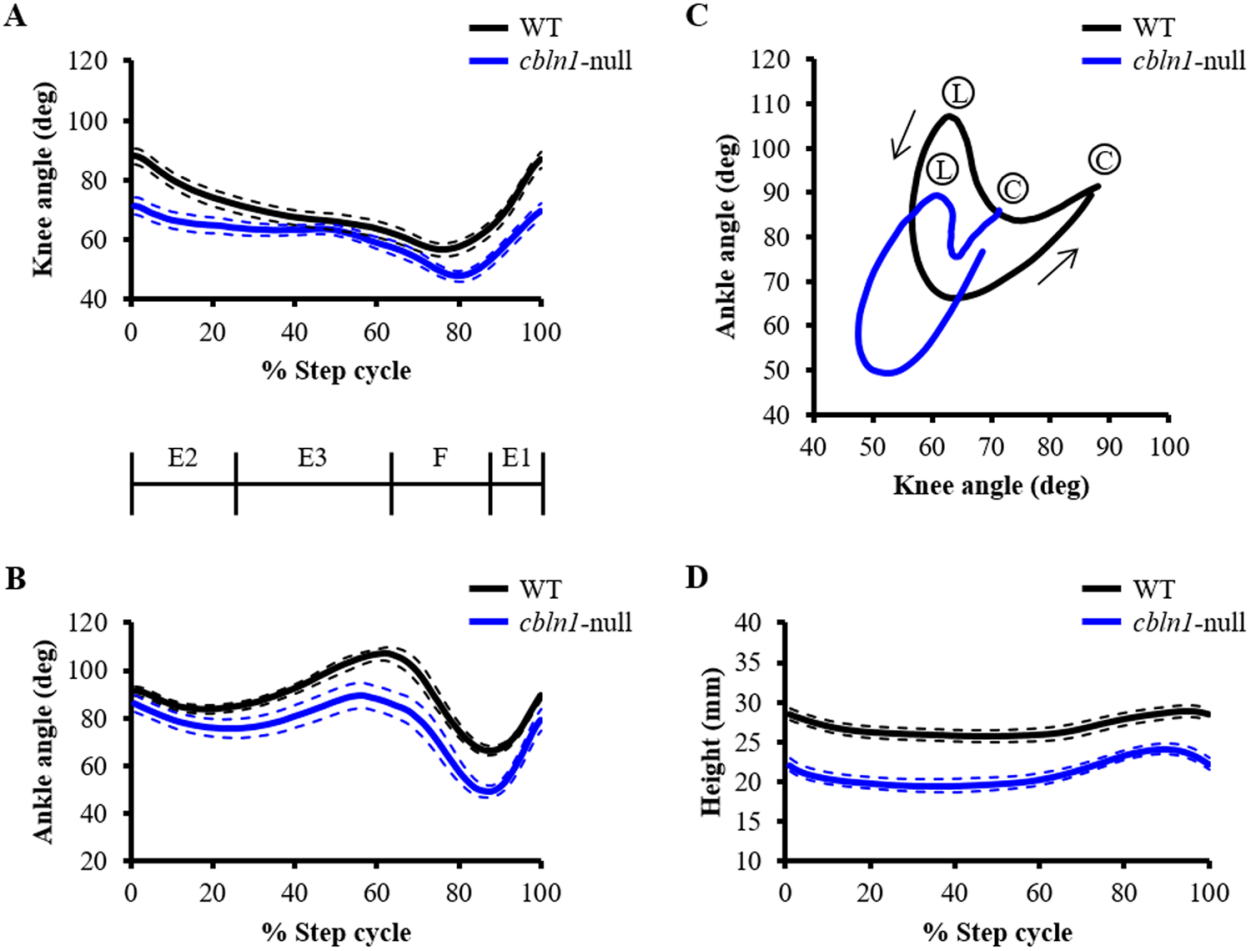
Kinematics of displacement during the step cycle. (**A,B**) Average angular displacements during the step cycles of wild-type (WT, black line) and *cbln1*-null (blue line) mice. Dotted lines indicate SEM. (**C**) Mean interjoint coordination patterns during the step cycle. Each line illustrates the averaged data for interjoint coordination patterns of the ankle and knee joints in wild-type (WT, black line) and *cbln1*-null mice (blue line). C: Foot contact at the beginning of the stance phase; L: foot lift at the beginning of the swing phase. Arrows indicate the direction of angular motions. (**D**) Average great trochanter height displacements during the step cycle in wild-type (black line) and *cbln1*-null (blue line) mice. Dotted lines indicate SEM.

Next, we performed a kinematic analysis of locomotion during walking at different subphases of the step cycle as described previously^17^ (Fig. 3). The knee and ankle joints of *cbln1*-null mice tended to be more flexed than those of wild-type mice, especially the knee at E2 and F phases (Fig. 3A) and the ankle at the transition phases from E3 to F and from F to E1 (Fig. 3B). The angles of excursion of the ankle relative to the knee are shown in Fig. 3C. The data indicates interjoint coordination between the ankle and knee joints. In the diagram, foot contact (C) is at the upper right and foot lift (L) is at the upper left. In wild-type mice, knee/ankle movements describe a crescent shape, similar to that reported previously in the normal rat^18^ and in our previous study in normal mice^6^. By contrast, knee/ankle movement in *cbln1*-null mice showed displacement of the curve with clearly distinguishable differences in contour patterns (Fig. 3C). This difference reflects the increased flexion of the knee and ankle joints and the reduced range of motion of the knee joint during the stance phase. Thus, this analysis of *cbln1*-null mice indicates increased discoordination between the knee and ankle compared to wild-type mice.

**Figure 4:**
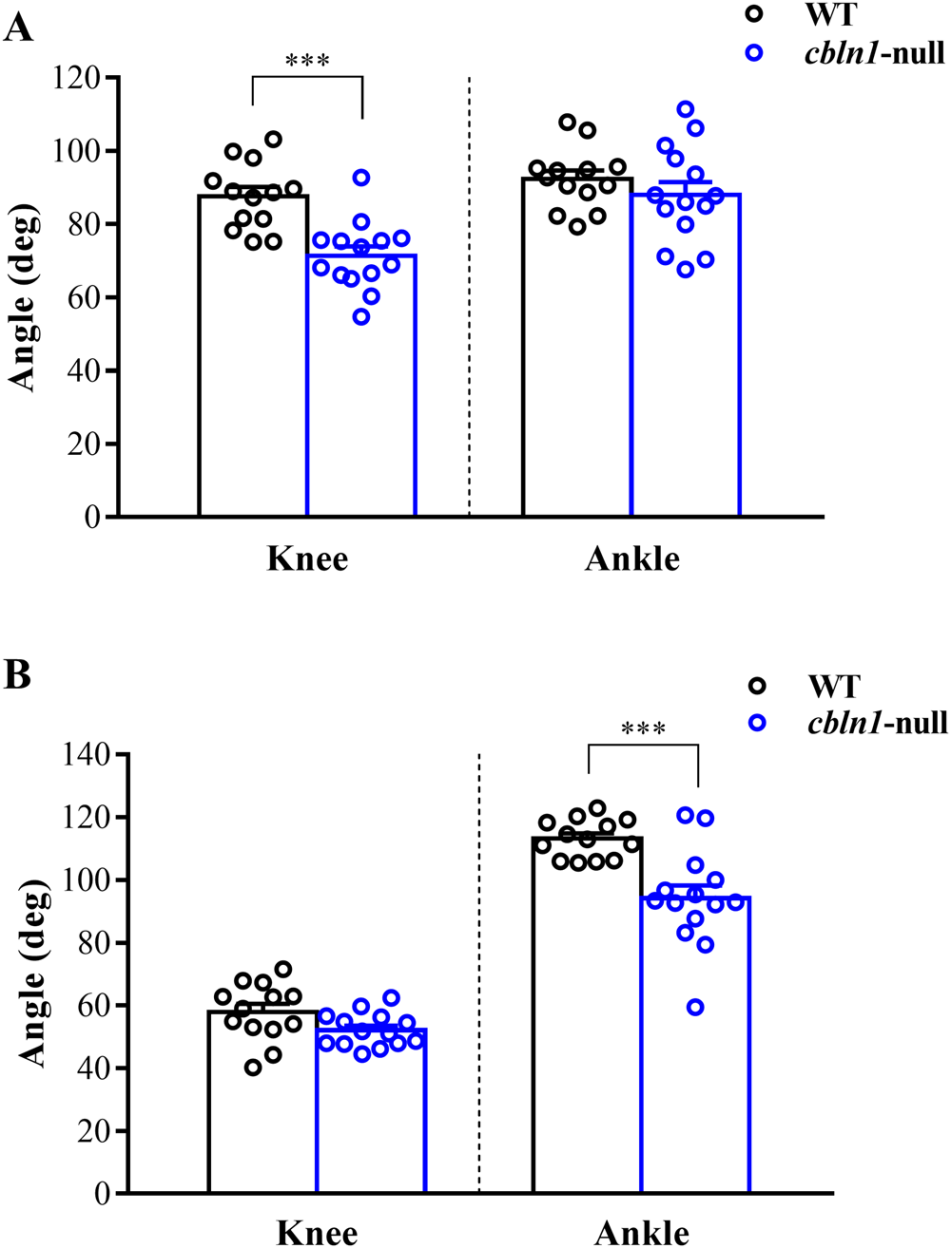
Joint angles at foot contact and foot lift. Knee and ankle joint angles at foot contact (**A**) and foot lift (**B**). ***p < 0.001.

Comparison of the displacement height of the great trochanter showed that it was lower in *cbln1*-null mice than in wild-type mice throughout the step cycle (Fig. 3D). Additionally, comparison of angle displacements showed that *cbln1*-null mice exhibited hyperflexion of their joint angles compared with wild-type mice around the phase transition. Therefore, we next compared knee and ankle angles at foot contact and foot lift in the two groups of mice (Fig. 4). At foot contact, the knee angle of *cbln1*-null mice was smaller than that of wild-type (*cbln1*-null = 71.4 ± 2.5 deg, wild-type = 87.6 ± 2.5 deg, t (25) = 4.573, *p* < 0.001, Fig. 4A). There was no significant difference in ankle angle between *cbln1*-null and wild-type mice (*cbln1*-null = 87.9 ± 3.5 deg, wild-type = 92.3 ± 2.3 deg, t (25) = 1.026, *p* = 0.3147, Fig. 4A). At foot lift, the knee angles did not differ between the two groups of mice (*cbln1*-null = 52.1 ± 1.4 deg, wild-type = 57.9 ± 2.6 deg, t (25) = 2.016, *p* = 0.0547, Fig. 4B). In contrast, the ankle angle of *cbln1*-null mice was significantly smaller than that of wild-type mice (*cbln1*-null = 94.1 ± 4.1 deg, wild-type = 113.2 ± 1.7 deg, t (25) = 4.151, *p < *0.001, Fig. 4B).

### Application of Cbln1 improves locomotor movements in *cbln1*-null mice

It was previously shown that injection of recombinant Cbln1 induced ultrastructural and electrophysiological changes to the function of PF synapses in the cerebella of *cbln1*-null mice and also reduced ataxia^15^. Here, we examined the effect of Cbln1 injection into the cerebellar vermis on locomotor movements in *cbln1*-null mice using a kinematic analysis. We have used the same method for injecting the recombinant Cbln1 as in the previous study^15^, because this method allows injected Cbln1 to distribute widely through the cerebellar lobules. We reconfirmed efficacy of the injection method by injecting a solution which contained Texas Red-dextran and confirmed that the dye was distributed widely in all cerebellar lobules (Supplementary Fig. 2).

**Figure 5:**
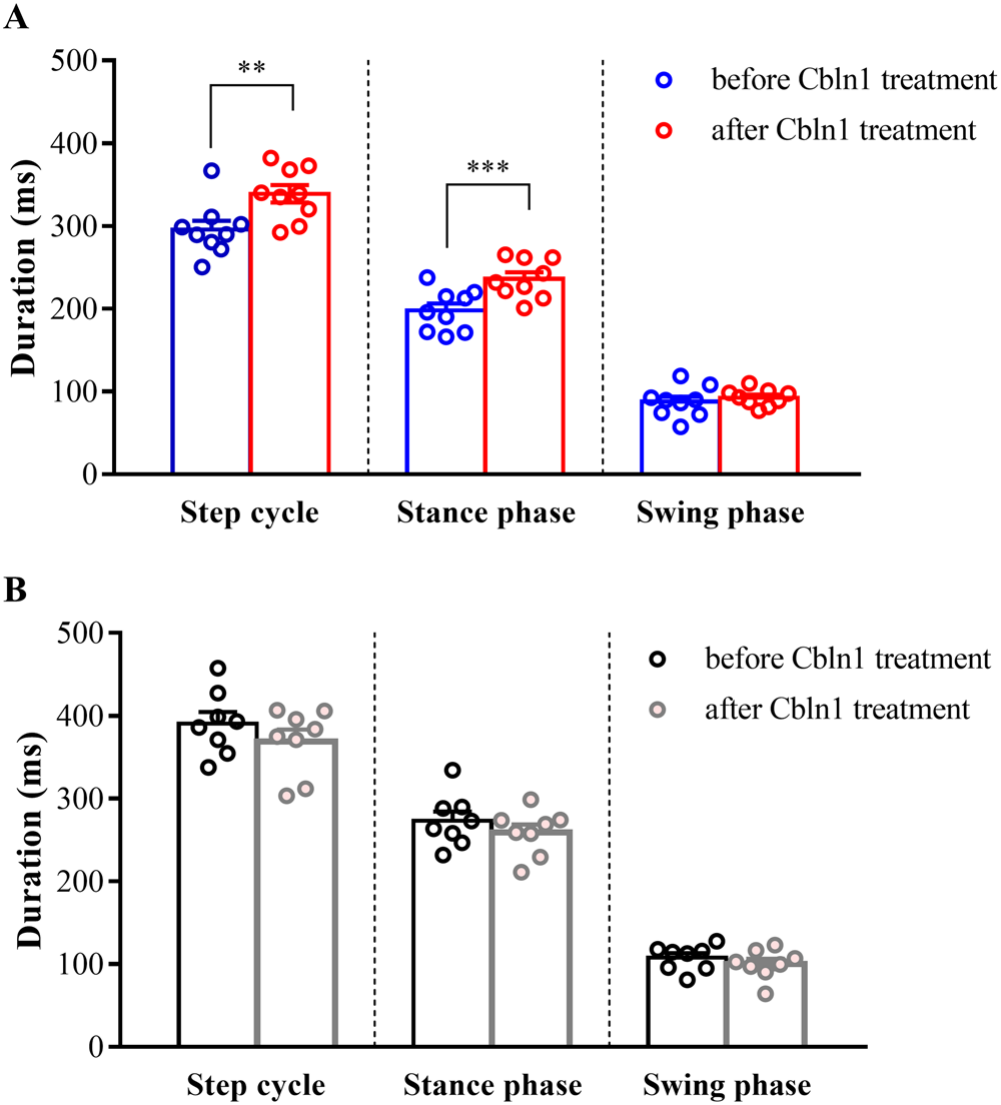
Average step cycle duration after injection of Cbln1. (**A**) Average step cycle, stance and swing phase durations in *cbln1*-null mice before (before treatment, blue) and after (Cbln1-treated, red) injection of Cbln1. (**B**) Average step cycle, stance phase and swing phase durations in wild-type mice before (before treatment, black) and after (Cbln1-treated, gray) injection of Cbln1. Differences in durations before and after injection of Cbln1 were tested using paired *t*-tests. **p < 0.01, ***p < 0.001.

The durations of the step cycle, stance phase and swing phase before and after injection of the protein are shown in Fig. 5. We found that the durations of the step cycle and stance phase increased after injection of Cbln1 into *cbln1*-null mice (step: before Cbln1 treatment = 295.6 ± 10.7 ms, after Cbln1 treatment = 338.8 ± 10.5 ms, t (8) = 4.146, *p* < 0.01; stance: before Cbln1 treatment = 197.9 ± 8.4 ms, after Cbln1 treatment = 236.1 ± 7.8 ms, t (8) = 5.403, *p* < 0.001; swing: before Cbln1 treatment = 87.7 ± 6.2 ms, after Cbln1 treatment = 92.7 ± 3.4 ms, t (8) = 0.7379, *p* = 0.4816, n = 9, paired *t*-test, Fig. 5A). However, injection of the protein had no significant effect on swing phase duration, and there were no significant differences in the durations of these various phases before and after injection of the protein into wild-type mice (step: before Cbln1 treatment = 390.7 ± 13.6 ms, after Cbln1 treatment = 369.1 ± 14.2 ms, t (7) = 0.8151, *p* = 0.4419; stance: before Cbln1 treatment = 273.1 ± 11.1 ms, after Cbln1 treatment = 259.1 ± 9.7 ms, t (7) = 0.7219, *p* = 0.4937; swing: before Cbln1 treatment = 107.6 ± 5.4 ms, after Cbln1 treatment = 100.1 ± 6.4 ms, t (7) = 0.7657, *p = *0.4689, n = 8, paired *t*-test, Fig. 5B). As a control experiment, we injected a mutant form of Cbln1, which lack two cysteine residues that are essential for hexamerization of Cbln1^11^. Previous study have shown that this mutant Cbln1 do not induce PF-PC synapse formation when injected into *cbln1*-null mice^15^. We found that there were no significant differences in the durations of the various phases before and after injection of a mutant form of the Cbln1 protein into wild-type and *cbln1*-null mice (Supplementary Table 1). These results indicate that injection of wild-type Cbln1 but not the mutant Cbln1 into the cerebellar vermis induced an increase in the duration of the step cycle and stance phase in the *cbln1*-null mice.

**Figure 6:**
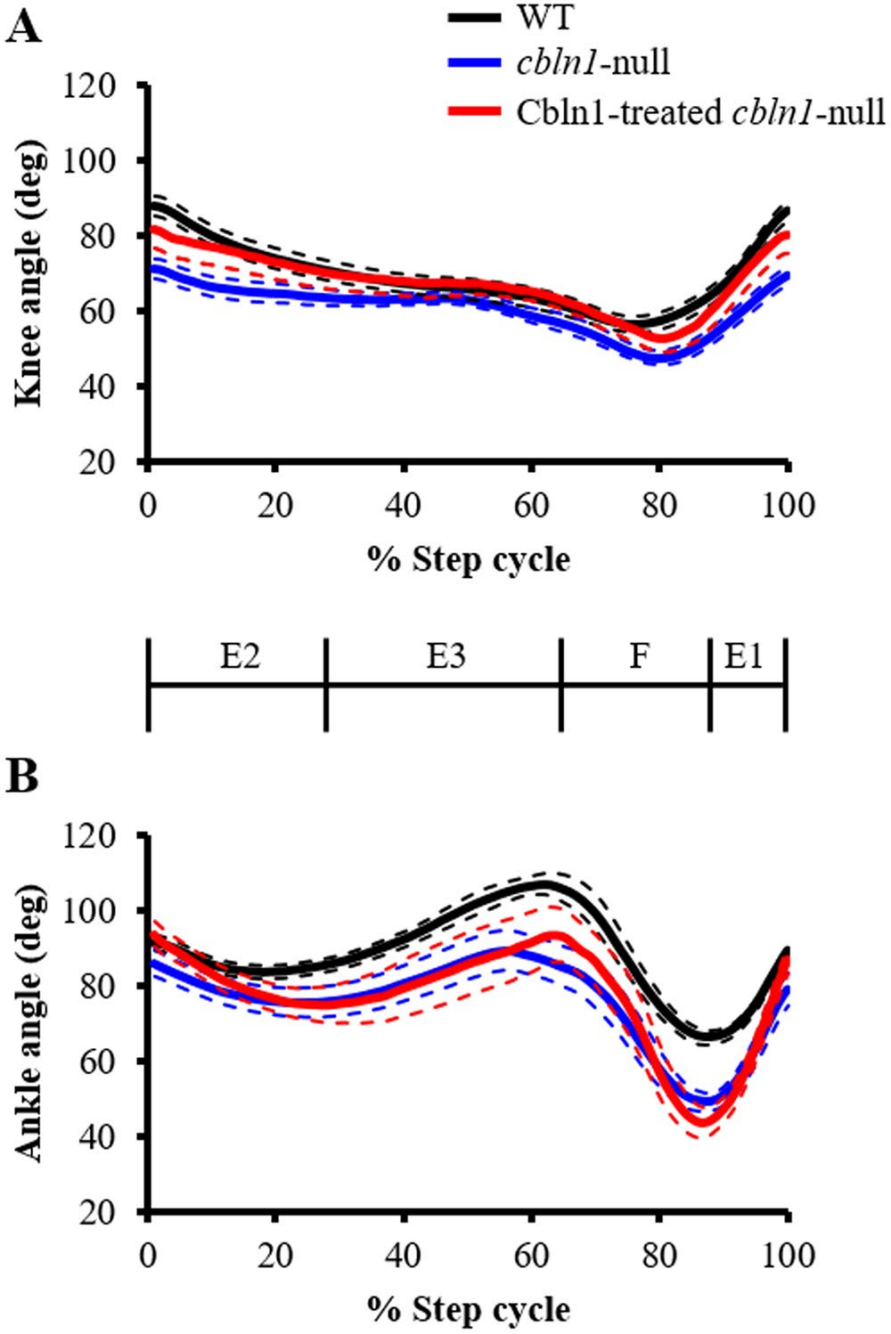
Joint angle displacement after injection of Cbln1. Average angular displacements after injection of Cbln1 into *cbln1*-null mice (Cbln1-treated *cbln1*-null, red line) during step cycles. (**A**) knee, (**B**) ankle.

**Figure 7:**
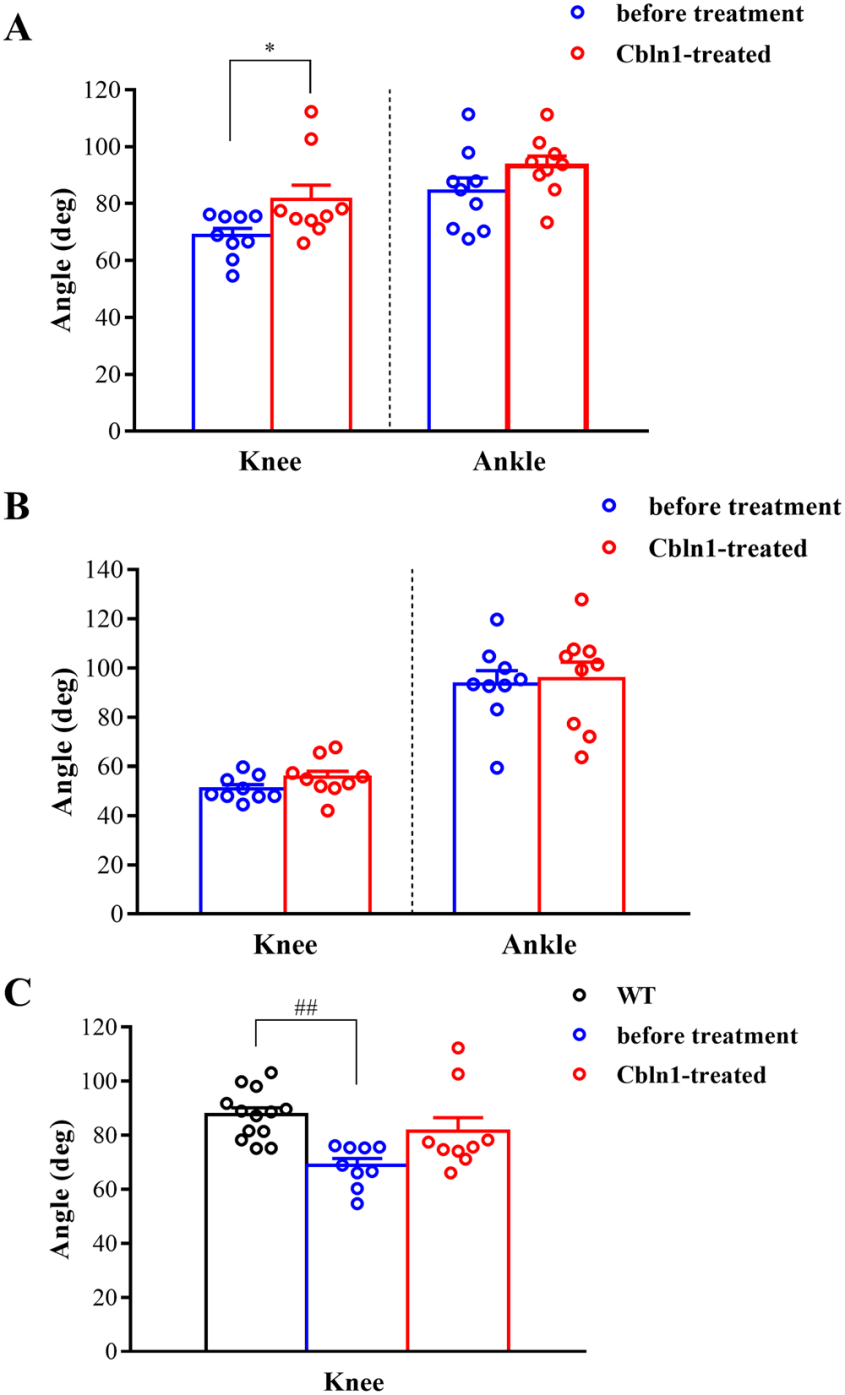
Angle at foot contact and foot lift after injection of Cbln1. Average knee and ankle angles at foot contact (**A**) and foot lift (**B**) in *cbln1*-null mice before (blue) and after (red) injection of Cbln1. (**C**) Knee angles of *cbln1*- null mice and wild-type mice after Cbln1 treatment. *p < 0.05 (paired-*t* test), ##*p* < 0.01 (one-way ANOVA, Tukey post-hoc test).

The angular displacements of joints in *cbln1*-null mice before and after injection of Cbln1 are shown in Fig. 6. For the ankle, although the timing of the peak angle around phase transition, from E3 to F, was closer to the wild-type after injection of Cbln1, the changes in ankle angles before and after injection were very small (Fig. 6B). However, with regard to the knee joint, the angular displacement of *cbln1*-null mice injected with Cbln1 was much closer to that of wild-type mice (Fig. 6A). There were no differences in knee or ankle angular displacements in wild-type mice injected with Cbln1 (Supplementary Fig. 3). These results indicate that injection of Cbln1 into the cerebellar vermis influenced angular motion, especially knee angle. To examine the angles around the phase transitions in more detail, the angles at foot contact and foot lift were compared in *cbln1*-null mice before and after injection of Cbln1 (Fig. 7). We found that injection of Cbln1 induced a significant difference in knee angle at foot contact in *cbln1*-null mice (at foot contact: before Cbln1 treatment = 68.8 ± 2.6 deg, after Cbln1 treatment = 81.4 ± 5.1 deg, t (8) = 2.899, *p* < 0.05, Fig. 7A; at foot lift: before Cbln1 treatment = 50.9 ± 1.7 deg, after Cbln1 treatment = 55.5 ± 2.6 deg, t (8) = 1.563, *p* = 0.1567, Fig. 7B). To determine whether Cbln1 treatment of null mice restored knee angle to the wild-type level, we compared knee angles in wild-type mice, untreated *cbln1*-null mice and Cbln1-treated *cbln1*-null mice (wild-type (n = 13) = 87.6 ± 2.5 deg; *cbln1*-null mice before Cbln1 treatment (n = 9) = 68.8 ± 2.6 deg; *cbln1*-null mice after Cbln1 treatment (n = 9) = 81.4 ± 5.1 deg; F (2, 28) = 0.3878, *p* < 0.01, Fig. 7C). In *cbln1*-null mice, knee angle at foot contact was less than that of wild-type mice. However, there was no significant difference in knee angles at foot contact between wild-type and Cbln1-treated *cbln1*-null mice; nor was there any difference between untreated and Cbln1-treated *cbln1*-null mice. These results indicate that Cbln1 treatment can restore the knee angle at foot contact in *cbln1*-null mice. For ankle angles, there was no significant difference between untreated and Cbln1-treated *cbln1*-null mice (at foot contact: before Cbln1 treatment = 84.4 ± 4.7 deg; after Cbln1 treatment = 93.3 ± 3.5 deg, t (8) = 1.73, *p* = 0.1218, Fig. 7A; at foot lift: before Cbln1 treatment = 93.5 ± 5.4 deg, after Cbln1 treatment = 95.6 ± 6.8 deg, t (8) = 0.293, *p* = 0.7770, Fig. 7B). Furthermore, there were no significant differences in knee and ankle angles before and after injection of the mutant Cbln1 into wild-type and *cbln1*-null mice (Supplementary Table 2).

**Figure 8:**
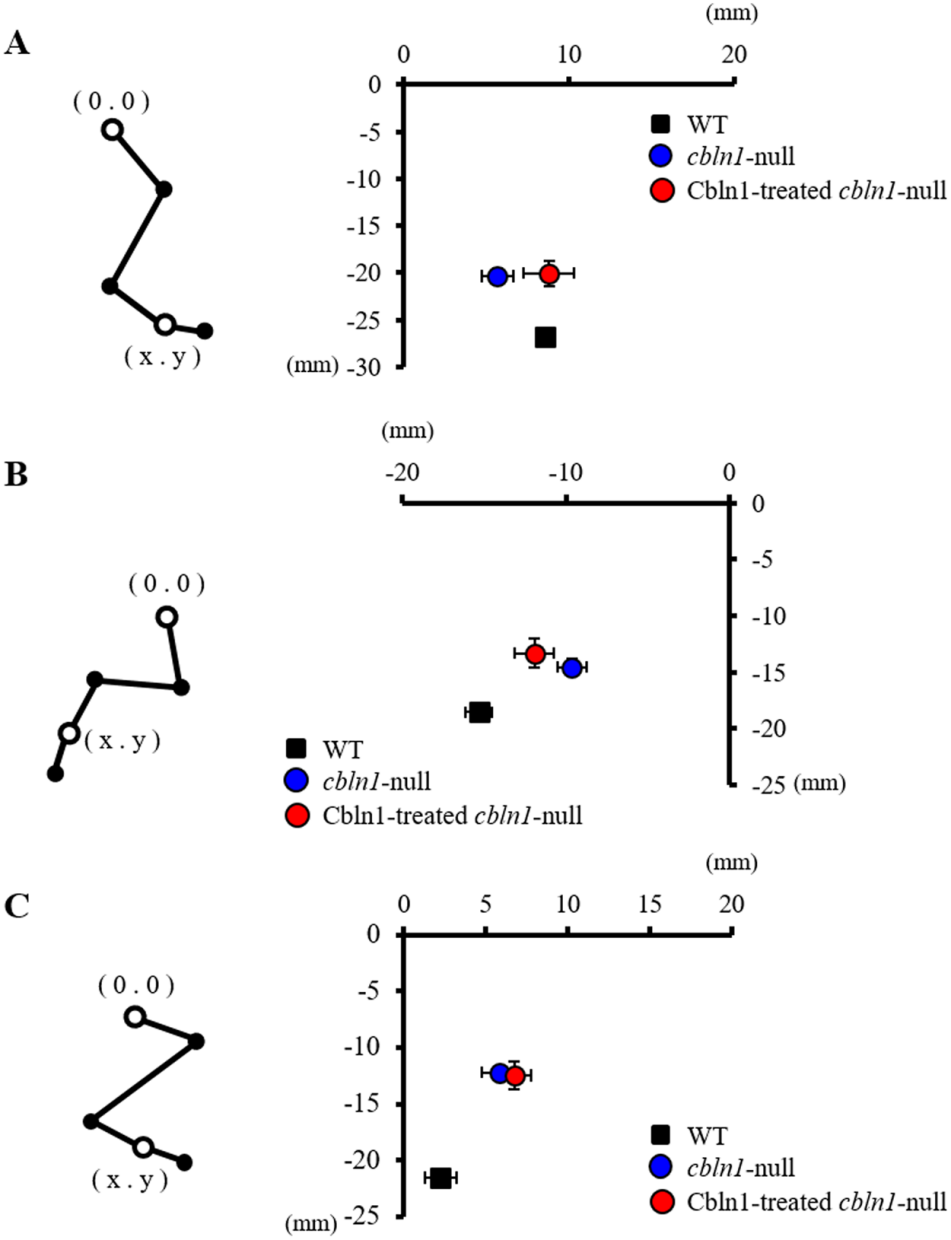
Position of the metatarsophalangeal (MTP) joint during the step cycle. Position of the MTP joint using the great trochanter as the origin. (**A**) Position of the MTP joint at foot contact. (**B**) Position of the MTP joint at foot lift. (**C**) Position of the MTP joint at maximal toe height. Error bars represent SEM.

The positions of the metatarsophalangeal (MTP) joint (see Fig. 2A), when the position of the great trochanter is regarded as the origin, in each of the three types of mice are shown in Fig. 8. The *cbln1*-null mice had a smaller distance between the great trochanter and the MTP joint than wild-type mice (Fig. 8), i.e., *cbln1*-null mice exhibited hyperflexion, a conclusion consistent with other observations made in this study (Figs 2D, 3 and 4). The *cbln1*-null mice placed their foot nearer to the tail than wild-type mice at foot contact. However, after injection of Cbln1, *cbln1*-null mice were able to position their foot more closely to that of wild-type mice (Fig. 8A). This result indicates that injection of the protein enabled *cbln1*-null mice to lift their limb forward in a more natural manner. Moreover, treated *cbln1*-null mice were able to lift their paw further back from the great trochanter compared to prior to treatment (Fig. 8B). However, the position of the MTP joint in the treated *cbln1*-null mice at maximal toe height was unchanged by injection of Cbln1 (Fig. 8C).

## Discussion

In this study, we performed a kinematic analysis of *cbln1*-null mice during treadmill locomotion and identified some features of abnormal locomotion in their gait. The duration of the step cycle, stance phase and swing phase were significantly reduced in *cbln1*-null mice compared to wild-type mice. The *cbln1*-null mice displayed excessive toe elevation caused by hyperflexion of the knee and ankle during the swing phase; they also showed discoordination of knee and ankle angles compared to wild-type mice. However, injection of Cbln1 into the cerebellum of *cbln1*-null mice caused the duration of the step cycle and stance phase to increase toward those of wild-type mice. After injection of Cbln1, the angular displacement of the knee in *cbln1*-null mice was similar to that of wild-type mice. We suggest that improved function of PF-PC synapses was the principal factor contributing to these improvements in locomotory movements in the *cbln1*-null mice.

In a previous study, we characterized gait ataxia in the mouse *hotfoot* mutation, *ho15J*^6^. The *ho15J* and *cbln1*-null mice show poor motor performance on a rotarod test^7,14^. In this test, mice have to maintain their balance on a rotating rod. Here, we found that *cbln1*-null mice showed discoordination of knee and ankle angles during locomotion. The *ho15J* mice also showed discoordination of these angles^6^. Thus, one of the reasons that mice of both genotypes show poor performance in a rotarod test is the discoordination of their knee and ankle angles during locomotion. Comparison of the ataxic gaits of *cbln1*-null and *ho15J* mice indicates that they show similar features; however, *cbln1*-null mice have a more severe ataxia than *ho15J* mice. Cbln1 is a ligand for GluD2^11^, and both proteins are essential for synapse integrity. The cerebellum of the *ho15J* mice is characterized by a reduction (~60%) in the number of PF-PC synapses compared with wild-type mice^6^, whereas *cbln1*-null mice show an approximately 80% reduction in PF-PC synapses compared with wild-type mice^14^. These observations indicate that the lower the number of PF-PC synapses then the greater the severity of ataxia. We presume that signals from PF-PC synapses are important contributors to the control of locomotion. The cerebellar neural circuit participates in the control of locomotion as part of the spinocerebellar loop. Motor commands from the spinal cord are carried by the ventral spinocerebellar tract (VSCT) and sensory information from the limbs is carried by the dorsal spinocerebellar tract (DSCT). The information in the VSCT and DSCT is conveyed through mossy fibers to granule cells and then to the cerebellar cortex. PCs integrate this information and then signal to neurons in the spinal cord through the cerebellar nuclei and brain stem neurons; in this way, the cerebellum controls spinal neuron activities^19,20^. DSCT neurons encode a combination of limb axis positions and movement velocities, whereas PCs encode this information independently. In particular, the DSCT mediates global parameters of hindlimb kinematics and is considered to be important for appropriate limb movement control during locomotion^21–24^. The PF-PC synapses have a central role for the sensorimotor information processing in the cerebellum during locomotion.

The *cbln1*-null mice showed hyperflexion of the knee and ankle joints during locomotion. In a study on cats, it was shown that cooling-induced reversible inactivation of the unilateral cerebellar intermediate cortex at lobules IV induces hyperflexion of the ipsilateral hip and ankle^25^. Moreover, unilateral cooling at lobules V of the intermediate region induces hyperflexion of the ipsilateral forelimb^25–27^. These observations indicate that the cerebellar cortex is responsible for the activities of limb muscles. The activity of vestibulospinal neurons increases during locomotion and their peak discharge usually occurs at the beginning of the stance phase of the ipsilateral hindlimb. This discharge is related to the activity of the extensor muscles^28,29^. Signaling from the rubrospinal neurons increases during locomotion and the peak discharge usually occurs during the swing phase of the contralateral hindlimb. This discharge is related to the activity of the flexor muscles^30^. Bulbar reticular formation is also concerned with the flexor muscles during the swing phase^29^. From an anatomical point of view, the cerebellar vermis projects to the reticular formation and lateral vestibular nuclei. The intermediate region projects to the red nuclei. It is possible that *cbln1*-null mice may show abnormal functions in these regions due to attenuation of the signal from the cerebellar cortex because of the reduced number of PF-PC synapses, which may result in loss of control of locomotor movement.

The *cbln1*-null mice showed a step cycle duration and stance phase duration closer to the wild-type after injection of Cbln1. As the swing phase showed no significant improvement after injection of the protein, then the overall improvement in temporal parameters of locomotion after injection of Cbln1 was due to the change in the stance phase (Fig. 5). The *cbln1*-null mice showed hyperflexion of the knee joint compared to wild-type mice at foot contact (Fig. 4A), and the angle of the joint increased after injection of Cbln1 (Fig. 7A). This result may indicate the *cbln1*-null mice could move their knee joint more easily during locomotion after treatment.

In the immature cerebellum, a PC is multiply innervated by CFs. During development, the elimination of supernumerary CFs occurs during the second and third postnatal weeks until a one-to-one relation between CFs and PCs is attained at approximately postnatal day 21. By contrast to the normal pattern of development, the cerebellum of *cbln1*-null mice has multiple CF innervations in the adult. It is unclear whether these multiple CF innervations induce gait ataxia. Some evidence against this comes from the observation that transgenic rescue of the abnormal phenotype of GluD2-null mice occurred in the presence of multiple CF innervation^31^. In the present study, we found a transient improvement in gait ataxia by an injection of the Cbln1 protein; our previous study showed multiple CF innervations were still present after Cbln1 treatment^32^. These observations suggest that multiple CF innervation might not strongly affect gait ataxia. In this study, parameters during the swing phase did not improve after Cbln1 treatment (Figs 5A and 7B). Hyperflexion of joint angles and excessive toe elevation during the swing phase occur after inactivation of the intermediate cerebellum^25–27,33^. In this study, we injected Cbln1 into the cerebellar vermis. Therefore, there is a possibility that the injected Cbln1 may not have diffused sufficiently through the intermediate cerebellum to improve gait ataxia. The improvement of gait ataxia corresponds with the time course of restoring PF-PC synapse formation^17^. We examined treadmill locomotion at 4 days after injection of Cbln1, allowing only a short time to acquire compensation. It is unlikely that the mice are able to improve muscle strength and induce muscle hypertrophy over such a short period. Therefore, we suggest that the reorganization of the PF-PC synapses is the leading cause of the rescue of the gait ataxia in *cbln1*-null mice.

## Methods

### Animals

Experiments were performed on *cbln1*-null mice (n = 14, 7–10 weeks of age, body weight 17.5 ± 0.7 g, Hirai *et al*.^14^). Wild-type C57BL/6 J mice (n = 13, 7–10 weeks of age, CREA Japan, Tokyo, Japan, body weight 22.8 ± 0.5 g) were used as controls. The animals were kept in a temperature-controlled room with a 12-h light/dark cycle (lights on from 08:00 to 20:00), and had ad lib access to food and water. The well-being of the mice was carefully monitored, and all efforts were made to minimize the number of animals used and any suffering in the course of the experiments. This study was approved by the Ethical Committee for Animal Experiments at the University of Tokyo, and was carried out in accordance with the Guidelines for Research with Experimental Animals of the University of Tokyo and the Guide for the Care and Use of Laboratory Animals (NIH Guide, revised in 1996).

### Locomotion recording

Before recording locomotion patterns, the mice were habituated to the treadmill apparatus and trained to walk on it. To enable observation of hindlimb movements, the fur on the right hindlimb of each animal was shaved under isoflurane gas anesthesia (3% for induction, 1–2% for maintenance). Circular reflective markers (2 mm diameters) were precisely placed on the shaved skin of the right hindlimb at the great trochanter, the knee, the lateral malleolus (ankle), the fifth metatarsophalangeal (MTP) joint, and a toe (Fig. 2A). The mice were allowed to recover completely from the anesthesia before being placed on the treadmill. The animals walked freely at different speeds (8, 16, and 24 m/min) imposed by the treadmill belt and their locomotor movements were recorded at 200 frames per second using a high-speed digital image camera system (HAS-220, DITECT, Inc., Tokyo, Japan). The captured images were stored electronically for later analysis.

### Motion analysis

Motion analysis was limited to the sagittal plane parallel to the direction of walking. Custom-designed image analysis software (DIPP-Motion Pro 2D, DITECT, Inc., Tokyo, Japan) was used to extract the two-dimensional coordinates of the various joint markers and to reconstruct a stick diagram representation of the right hindlimb. Due to skin slippage above the knee joint during walking, the actual knee position was corrected by triangulation from the position of the hip and ankle joint, using the measured lengths of the femur and tibia. In this study, we defined a step cycle as always having a phase in which the mouse supported its body weight with both hindlimbs (bisupport phase). If there was no bisupport phase in the observed cycle, then we excluded the data from analysis because the mouse was unable to walk. Ten step cycles were used for temporal parameters and kinematic analysis of each mouse. The step cycle can be divided into the stance phase and the swing phase. The stance phase is defined as starting when the foot touches the treadmill belt and as ending when the foot lifts from the belt. The swing phase is defined as starting at the moment when the animal lifts its foot from the treadmill belt and as ending when the foot comes back into contact with the treadmill belt.

To analyze the angular excursions of the knee and ankle during a cycle, the step cycle duration was normalized, and cubic-spline interpolation was applied to the original data on the joint angles of the knee and ankle to obtain 100 samples per step cycle regardless of their duration using MATLAB software (MathWorks Inc., Natick, MA).

### Injection of recombinant Cbln1

Recombinant Cbln1 was prepared as described previously^15^. In brief, the medium from cultures of human embryonic kidney cells that express Cbln1 was collected 48 hours after the transfection by the calcium phosphate method. The culture medium was concentrated using spin columns with ultrafiltration membranes and then dissolved in PBS to a final concentration of 1–4 μg/μl. Recombinant Cbln1 was injected into the subarachnoidal space in the cerebellar vermis (1 μl/g body weight). Mice were briefly anesthetized with isoflurane (3% for induction, 1–2% for maintenance), a small hole in the occipital bone was made with a dental drill, and the dura matter was ablated. A microsyringe needle was inserted into the surface of the cerebellar vermis, and the solution containing recombinant Cbln1 was injected into the subarachnoidal space at a rate of 40 μl/h with a Hamilton syringe. The effect of Cbln1 on motor performance in rotarod tests was examined in a previous study^15^. The time on the rotarod reached a peak at 2 days after injection and the effect lasted until day 4; thereafter, the performance gradually worsened to day 7. Taking these results into consideration, we analyzed locomotion at 4 days after Cbln1 injection to obtain the maximum effect. Mice of each genotype were divided into two groups: one received the Cbln1 injection (*cbln1*-null mice, n = 9; wild-type mice, n = 8); the other received an injection of a mutant form of the Cbln1 protein (*cbln1*-null mice, n = 5; wild-type mice, n = 5). The mutant form of the Cbln1 protein was generated by replacing cysteine 34 and cysteine 38 with serines. This mutant Cbln1 was previous shown to lack S-S bondages essential for hexamerization and lack capacity to induce PF-PC synapse formation^15^.

### Statistical analysis

All data were analyzed using the Statistical Package for Social Sciences (SPSS, Japan, Inc.,Tokyo, Japan) version 14.0 using Student’s *t*-test, Welch’s test, paired *t*-test, one-way ANOVA, and the Tukey post hoc test. The level of statistical significance for variables was set at *p < 0.05, **p < 0.01, ***p < 0.001. Data are expressed as means ± standard error of the mean.

## Electronic supplementary material

Supplementary information
