# Supplementary material for: Improvement of cerebellar ataxic gait by injecting Cbln1 into the cerebellum of cbln1-null mice
Source: Sci Rep. 2018 Apr 18;8:6184. doi: 10.1038/s41598-018-24490-0 (PMC5906462; doi:10.1038/s41598-018-24490-0)

**Supplementary information**

**Improvement of cerebellar ataxic gait by injecting Cbln1 into the cerebellum of *cbln1*-null mice**

Eri TAKEUCHI^1^, Aya ITO-ISHIDA^2^, Michisuke, YUZAKI^2^ and *Dai YANAGIHARA^1^

^1^Graduate School of Arts and Sciences, The University of Tokyo, 3-8-1 Komaba, Meguro, Tokyo 153-8902, Japan

^2^Department of Physiology, School of Medicine, Keio University, 35 Shinanomachi, Shinjuku, Tokyo 160-8582, Japan

To whom correspondence should be addressed to Dai Yanagihara, Ph.D.

Tel: + 81-3-5454-6857

E-mail: dai-y@idaten.c.u-tokyo.ac.jp


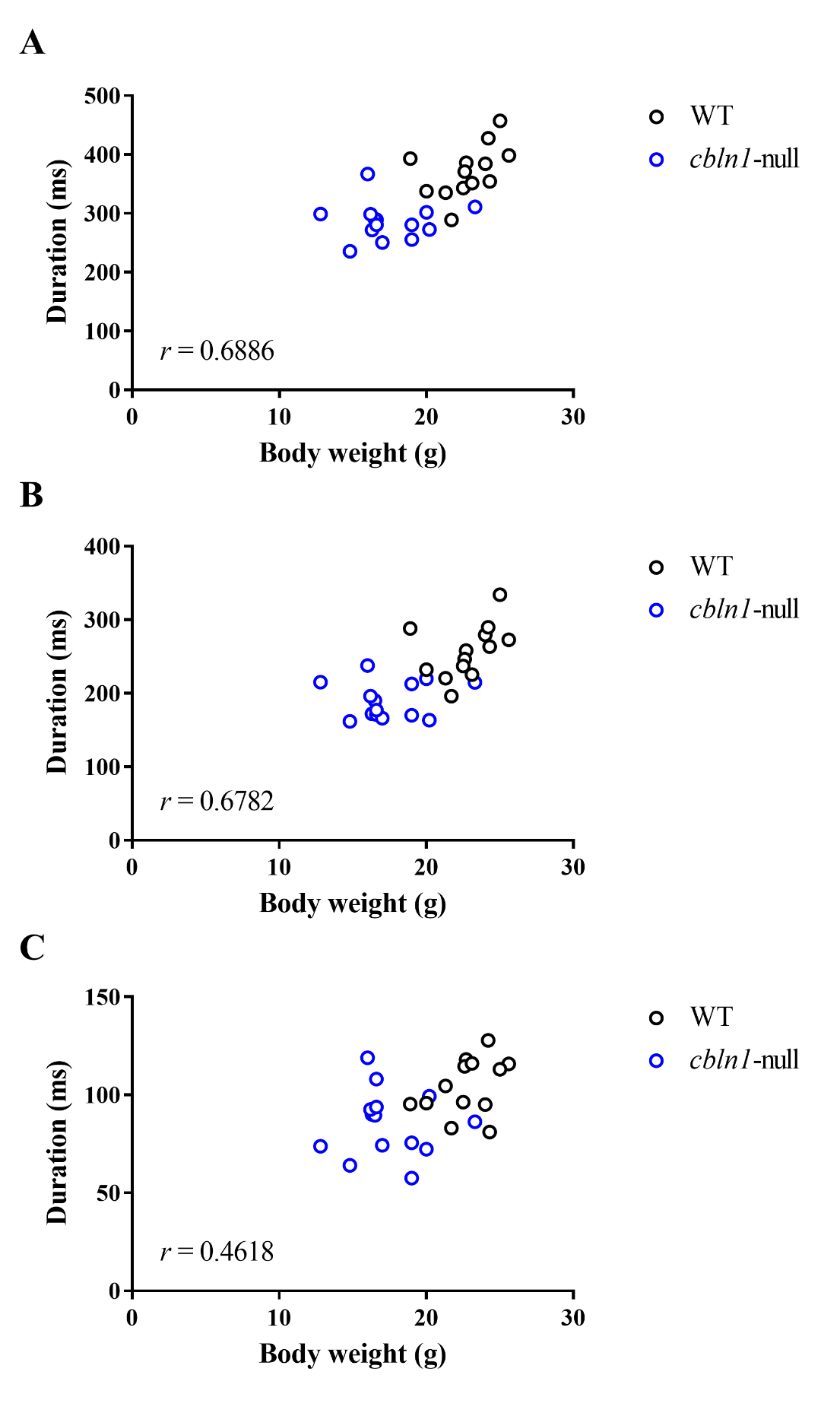


**Supplementary Fig. 1. Correlation between temporal parameters and body weight.**

The relationships between the various temporal parameters were investigated by correlation analyses. The data were first shown to have a normal distribution using D’Agostino-Pearson omnibus tests. Then, Pearson’s correlation or Spearman’s rank correlation analyses were performed. We identified a correlation between step cycle duration and body weight (A), between stance phase duration and body weight (B) and between swing phase duration and body weight (C). Positive correlations were found for all individuals (Step: *r­* = 0.6886, *p* < 0.0001; Stance: *r­* = 0.6782, *p* < 0.001; Swing: *r­* = 0.4618, *p* < 0.05). However, there was no correlation within the wild-type mouse group (Step: *r­* = 0.4822, *p* = 0.0952; Stance: *r­* = 0.4317, *p* = 0.1408; Swing: *r­* = 0.3668, *p* = 0.2177) or within the *cbln1*-null mouse group (Step: *r­* = -0.01101, *p* = 0.9728, Spearman’s rank correlation coefficient; Stance: *r­* = 0.1074, *p* = 0.7147; Swing: *r­* = -0.04509, *p* = 0.8784).


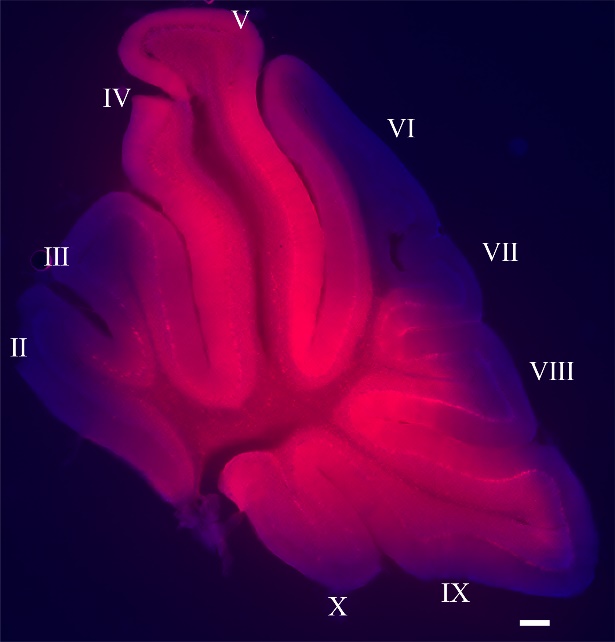


**Supplementary Fig. 2. Diffusion of injected dye.**

Representative sagittal section of the diffusion area stained with Texas Red-dextran (red) and DAPI (blue). Scale bars represent 200 μm.

A previous study showed that Cbln1 injected into the cerebellum spreads to all cerebellar lobules (Ito-Ishida et al., 2008, Figure 5A). In the present study, we used the same injection protocol as in the previous study. Additionally, we conducted a small experiment to confirm the spread of the injectate using 5% Texas Red-conjugated 3K dextran. After injection of Texas Red-dextran into the midline of the cerebellar vermis (1 μl/g body weight), the mice were perfused with 4% paraformaldehyde. The brains were removed and post-fixed in 4% paraformaldehyde. Sagittal sections (80 μm) were cut using a vibratome. The sections were mounted using antifade reagent with DAPI.


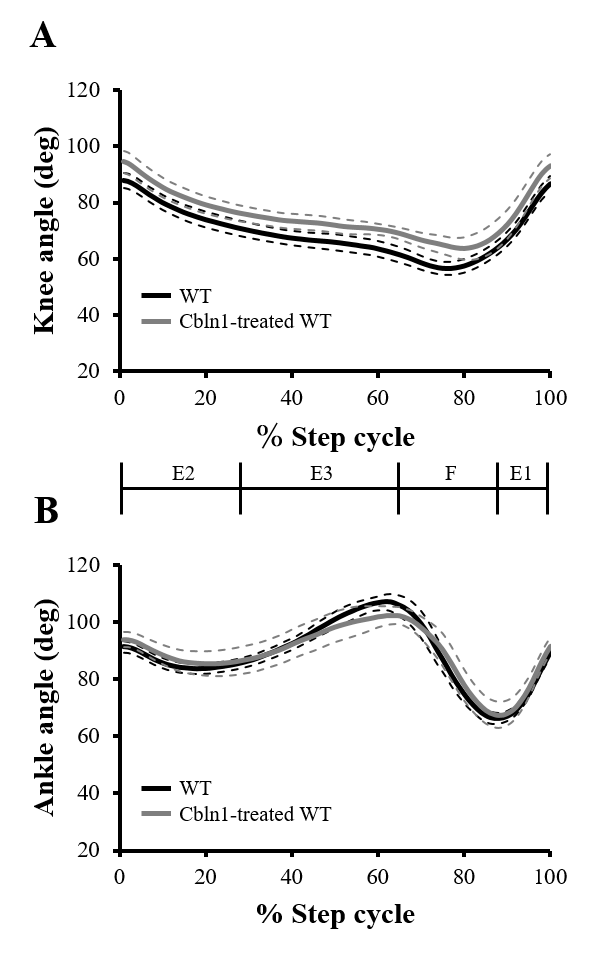


**Supplementary Fig. 3. Joint angle displacement of WT after injection of Cbln1.**

Average knee and ankle angular displacements during step cycles. Angular displacements after injection of Cbln1 into wild-type mice (Cbln1-treated WT, gray line). Knee angle and ankle angle of the step cycle were normalized to obtain 100 samples per step cycle. Dotted lines indicate SEM. (A) Knee angles, (B) ankle angles.


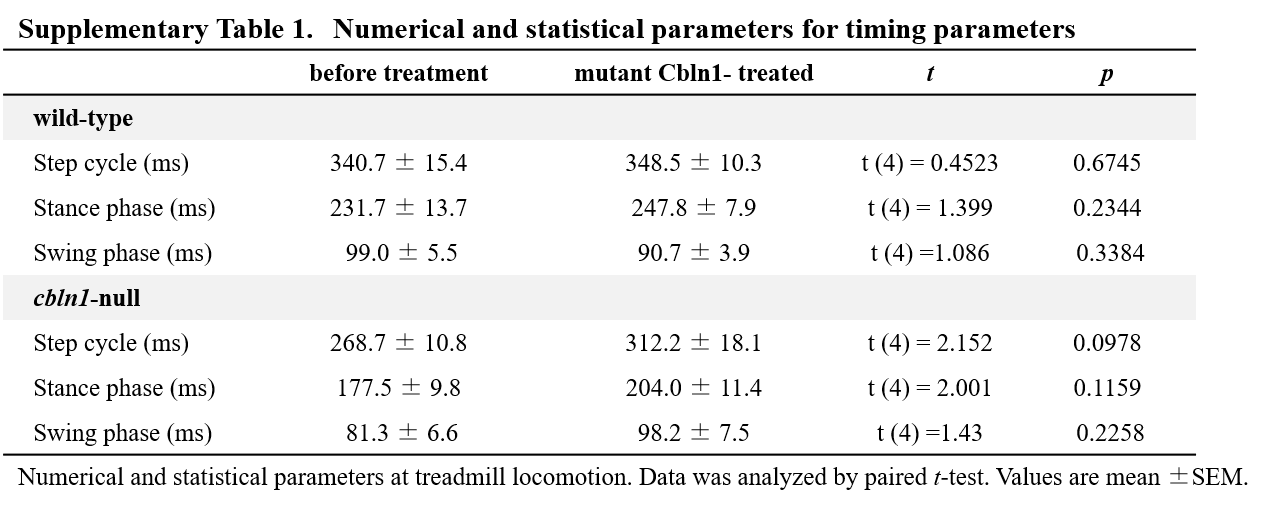


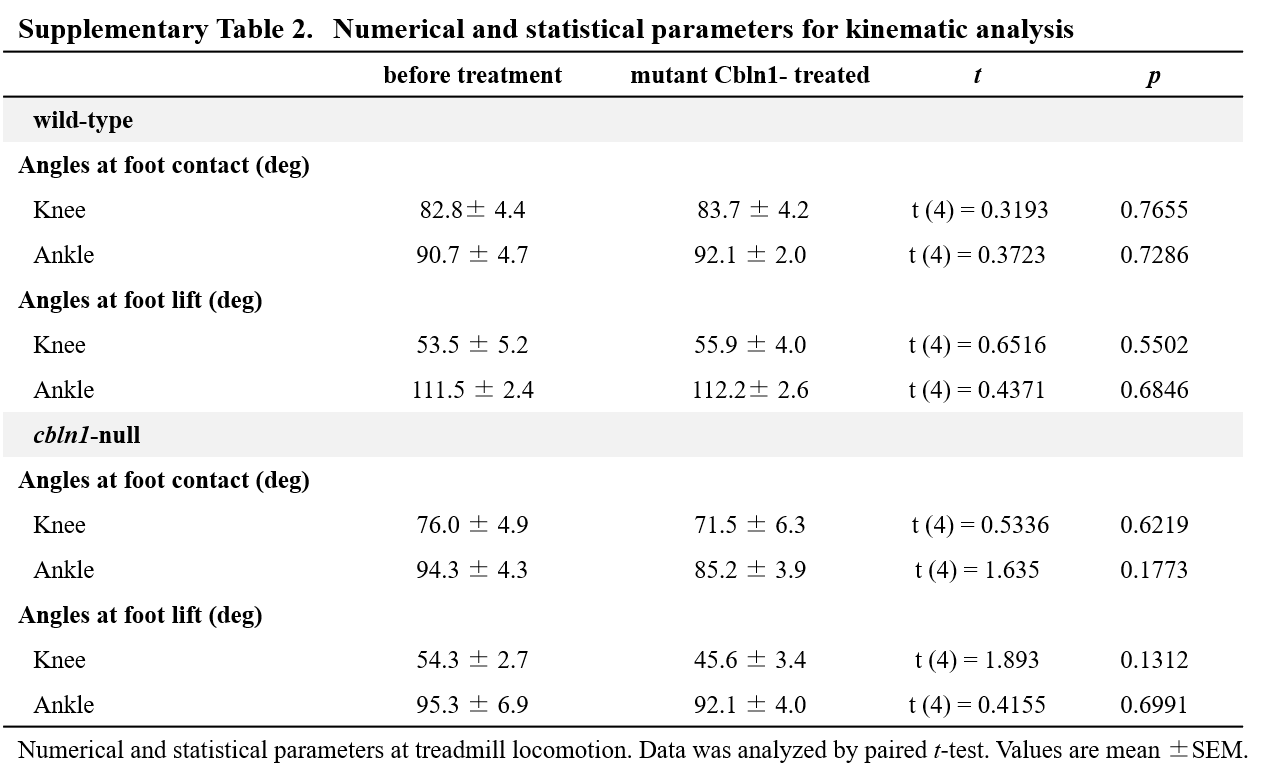

Supplement: Supplementary file 1 — Supplementary information [file 41598_2018_24490_MOESM1_ESM.docx]
